# Spatial, temporal and demographic distribution characteristics of adenomyosis symptom clusters from the perspective of traditional Chinese medicine: a multicenter cross-sectional study in China from 2020 to 2022

**DOI:** 10.3389/fendo.2025.1605310

**Published:** 2025-08-07

**Authors:** Xin Wang, Yu Tao, YuDan Fu, XinChun Yang, RuiHua Zhao

**Affiliations:** ^1^ Guang’anmen Hospital, China Academy of Chinese Medical Sciences, Beijing, China; ^2^ Clinical Medical College, Beijing University of Chinese Medicine, Beijing, China

**Keywords:** adenomyosis, spatial, temporal, demographic, symptom clusters

## Abstract

**Objective:**

This study aimed to explore the differences in symptom clusters of adenomyosis (AM) patients across spatial, temporal, and age-stratified dimensions based on the theory of “Treatment in Accordance with Three Categories of Etiologic Factors”.

**Methods:**

A cross-sectional study was conducted in China from 2020 to 2022, involving 1,816 AM patients. Univariate analysis, symptom co-occurrence matrices, and hierarchical clustering were used to compare clinical and symptom cluster characteristics across different latitudes (low latitude area: 691 cases vs. high latitude area: 1,125 cases), seasons (warm: 652 cases vs. cold: 473 cases), and age groups (<40 years: 895 cases vs. ≥s. years: 921 cases).

**Results:**

AM symptom clusters exhibited significant geographical, seasonal, and age-related variations, with “Blood Stasis Syndrome” as the core pathological mechanism. In the spatial dimension, the high latitude region exhibited a “syndrome of Cold Coagulation” characterized by cold intolerance (co-occurrence frequency: 3,973), treated by warming Yang and dispersing cold. The low latitude region displayed a “Spleen-Qi Deficiency and Blood Stasis” marked by fatigue (co-occurrence frequency: 2,492), treated by invigorating the spleen and supplementing Qi. In the temporal dimension, during cold seasons, cold intolerance (co-occurrence frequency: 2,029) reinforced the “Cold Coagulation”, treated by warming Yang and dispersing cold. In warm seasons, sweating had the highest co-occurrence frequency (2,054), suggesting a “Dampness” component, treated by invigorating the spleen to resolve dampness. In the age dimension, younger patients exhibited a “Cold Coagulation and Blood Stasis with Liver Qi Stagnation” with cold intolerance as the core symptom (co-occurrence frequency: 3,171), accompanied by irritability (39.66%) and premenstrual or menstrual irritability (34.30%), treated by warming Yang, dispersing cold, and soothing the liver to regulate Qi. Elder patients displayed a “Qi Deficiency and Blood Stasis” pattern with fatigue as the core symptom (co-occurrence frequency: 2,121), accompanied by menorrhagia (40.28%), treated by supplementing Qi and nourishing blood.

**Conclusion:**

Integrating TCM theory with data mining techniques, this study established a “time-space-human” three-dimensional syndrome differentiation model for AM, providing a critical framework for advancing precision medicine in TCM gynecology.

## 1 Introduction

Adenomyosis (AM) is a challenging gynecological disorder characterized by the abnormal growth of endometrial glands and stroma within the myometrium (1). Imaging-based diagnostic data indicate that approximately 20prox of women are affected by AM, with a higher prevalence among those aged 36–40 years (2). Clinical manifestations of AM include uterine enlargement, firm texture, globular configuration, and symptoms such as dysmenorrhea, chronic pelvic pain, heavy menstrual bleeding, and infertility (3), all of which significantly impair patients’ quality of life and reproductive health. Conventional Western medical treatments primarily focus on suppressing the hypothalamic-pituitary-gonadal axis (4), while they are often associated with adverse effects and high recurrence rates, failing to meet patients’ expectations for improved quality of life and fertility preservation (5). Consequently, exploring novel therapeutic approaches for AM remains a critical research priority in obstetrics and gynecology. Traditional Chinese Medicine (TCM) has demonstrated notable efficacy in alleviating clinical symptoms, controlling lesion progression, preventing recurrence, and supporting fertility in AM patients, with the advantages of minimal side effects, preservation of normal menstrual physiology, and compatibility with fertility goals (6). Harnessing the therapeutic potential of TCM may offer an effective solution to this clinical challenge.

“Treatment in Accordance with Three Categories of Etiologic Factors” represents one of the fundamental theories of TCM, encompassing “seasonal adaptation,” “geographical consideration,” and “individualized treatment.” This theory reflects the holistic philosophy of TCM, which views humans as an integral part of nature and society (7). Clinicians are expected to formulate personalized treatment strategies by comprehensively analyzing symptoms in the context of seasonal variations, regional characteristics, and individual constitutions. However, TCM syndrome differentiation is often subjective, ambiguous, and complex, with most clinical information being qualitative rather than quantitative (8). This poses challenges in elucidating the distribution patterns and characteristics of disease syndromes. “Symptom cluster” refers to a group of three or more interrelated symptoms, which is the basis of TCM diagnosis and classification (7). It is suggested that symptom cluster can simplify the components of the model and improve the accuracy of diagnosis (9). For instance, a study on stable angina pectoris revealed that while chest pain, fatigue, and dyspnea all impacted quality of life, fatigue was a more robust predictor than pain (10). Similarly, precise identification of core symptom clusters can enhance the efficiency of TCM syndrome differentiation. TCM emphasizes that people are a whole, and syndrome differentiation and treatment need to consider the symptoms of the whole body, not just the specific symptoms of the disease (11). Extracting the core symptoms from the complex systemic symptoms is helpful for accurate syndrome differentiation. An analysis of 1,741 clinical cases successfully extracted three core symptom clusters corresponding to Heart Qi Deficiency, Heart Yang Deficiency, Heart-Spleen Qi Deficiency, and Heart Fire Hyperactivity syndromes (12). The accuracy of syndrome differentiation is closely related to the curative effect of traditional Chinese medicine, and accurate syndrome differentiation is helpful to choose suitable Chinese medicines (13).

Therefore, this cross-sectional study investigated the current status of AM diagnosis and treatment in China from 2020 to 2022. By analyzing clinical data across seasons, regions, and age groups, we aimed to delineate the temporal, spatial, and demographic distribution characteristics of symptom clusters in Chinese AM patients. The findings provide empirical support for the “Treatment in Accordance with Three Categories of Etiologic Factors” theory, thereby reinforcing the role of TCM in AM management.

## 2 Materials and methods

### 2.1 Study population

This multicenter cross-sectional study was approved by the Ethics Committee of Guang’anmen Hospital, China Academy of Chinese Medical Sciences (Approval No. 2020-40-KY). All participants provided written informed consent before completing the questionnaire. This study was conducted in 45 hospitals in 19 provinces of China from 2020 to 2022, and the clinical data of AM patients were collected by trained clinicians. Inclusion criteria: (1) Diagnosis of AM confirmed by ultrasound, MRI, or pathology. The ultrasound diagnosis of AM is based on the Morphological Ultrasound Assessment (MUSA) consensus criteria and the Chinese Expert Consensus on the Diagnosis and Treatment of AM. The key diagnostic features of adenomyosis on ultrasound include: ①Asymmetric myometrial thickening, especially in the posterior wall and fundus of the uterus. ②Cystic lesions within the myometrium. ③Hyperechoic islands within the myometrium. ④Radiating, fan-shaped shadowing within the myometrium, characterized by alternating hypoechoic and hyperechoic linear stripes traversing the uterine wall. ⑤Subendometrial linear or bud-like hyperechoic signals. ⑥Translesional vascular signals, i.e., the presence of vascular signals crossing the lesion. ⑦Irregular junctional zone (JZ). ⑧Discontinuous JZ. A tentative diagnosis of AM can be made if two or more of the above signs are present. Since the cross-sectional survey was conducted from 2020 to April 2022, we used the 2018 FIGO-approved ultrasound diagnostic criteria for adenomyosis. According to the Chinese Expert Consensus on the Diagnosis and Treatment of Adenomyosis, the key features for MRI diagnosis are: the presence of ill-defined, low-signal-intensity lesions within the uterus, high-signal-intensity lesions on T2-weighted images, and an enlarged endometrial-myometrial junctional zone, greater than 12mm. All our imaging examinations (ultrasound and MRI) were performed by experienced sonographers and radiologists. Based on the imaging diagnosis, gynecologists who had undergone rigorous training made the clinical diagnosis of adenomyosis according to clinical symptoms (①progressive worsening of dysmenorrhea; ② menorrhagia and/or prolonged menstrual periods; ③ infertility) and signs (bimanual or trimanual examination often reveals an enlarged uterus, spherical in shape, or with localized nodular protrusions, hard in texture, and tender to palpation, with more pronounced tenderness during menstruation (Bimanual or Trimanual examination often reveals an enlarged uterus, spherical in shape, or with localized nodular protrusions, hard in texture, and tender to palpation, with more pronounced tenderness during menstruation, and the uterus is often in a retroverted position with poor mobility). (2) Cognitively capable of independently completing the questionnaire; (3) Signed informed consent;(4) Questionnaires meeting quality control standards. Exclusion criteria:(1) Patients with neuropsychiatric disorders or inability to cooperate; (2) Questionnaires with obvious logical inconsistencies or >20% missing data.

### 2.2 Data collection

The questionnaire in this study investigated six categories of self-reported symptoms in AM patients, including menstrual symptoms, non-menstrual symptoms, sleep-related symptoms, gastrointestinal symptoms, limb symptoms, and psychological symptoms. The frequency of each symptom occurrence was counted in Excel. Patients’ geographical locations were determined by their residential addresses provided in the questionnaire, while seasons were classified according to the survey completion dates. Ages were calculated from birthdates. Additional collected data included patients’ height, weight, education level, income, occupation type, parity history, abortion history, AM surgical history, and treatment needs.

Subsequently, based on geographical locations, patients in this cross-sectional study were divided into high latitude region (Chinese provinces between 30°N and 60°N) and low latitude region (Chinese provinces between 0°N and 30°N) to analyze the spatial distribution characteristics of AM symptom clusters. Second, according to China’s meteorological features where high-latitude regions have significant temperature variations, patients from high-latitude areas were further stratified into warm season (March to August) and cold season groups (September to February of the following year) to examine the temporal distribution patterns of AM symptom clusters. Seasonal division follows China’s official seasonal classification standards (https://www.zgbk.com/ecph/words?SiteID=1&ID=47892&Type=bkzyb/): March to August are defined as spring-summer seasons with warm-hot climates, while September to February of the following year are autumn-winter seasons with cool-cold climates. Finally, patients were categorized into <40 years and ≥nd years groups based on average age to explore the age-related distribution characteristics of AM symptom clusters.

### 2.3 Data analysis

The incidence rate of each symptom was calculated as: (frequency of symptom occurrence/total number of participants) ×100%. By computing the co-occurrence frequency of each symptom pair within the study, we aggregated the discrete symptom data into a cohesive whole and constructed a symmetric matrix. Each element in this matrix represented the number of simultaneous occurrences of the corresponding symptom pair. For data analysis and visualization, we utilized R software (version 4.1.2). Incidence rates of symptoms were displayed through hierarchical network diagrams and bar charts. The co-occurrence matrix was visualized and classified using heatmaps and hierarchical clustering methods to identify core symptom clusters. The top 10 symptoms within each cluster that are most closely associated with other symptoms and designate these as the core symptoms. As the symptoms pointing to Blood Stasis Syndrome (dysmenorrhea and menstrual clot) are unique symptoms of AM, besides them, the symptoms most frequently associated with other core symptoms are considered as their represent symptoms (7). Continuous data were presented as mean ± standard deviation. For normally distributed data, one-way ANOVA was employed for intergroup comparisons, while the Kruskal-Wallis test was used for non-normally distributed data. Categorical data were described as frequencies (percentages), with intergroup comparisons performed using chi-square tests or Fisher’s exact tests. P-value <0.05 was considered statistically significant.

### 2.4 Judgment standard of TCM syndrome

In this study, TCM syndromes have adopted standardized and unified translations, accompanied by detailed definitions and modern scientific explanations.

Qi, a key concept in TCM, is indeed an abstract idea for Western scholars. TCM generally holds that Qi, often described as vital energy, is believed to flow through meridians or channels in the body, nourishing and supporting the organs. Qi Deficiency can be understood as energy insufficiency, while Qi Stagnation refers to abnormal energy flow.

A study has described “systemic Qi Deficiency” and “visceral Qi Deficiency”, where systemic Qi deficiency is abbreviated as “Qi Deficiency”, and a common example of visceral Qi deficiency is “Spleen-Qi Deficiency”. The same applies to “Qi Stagnation”: as a systemic pattern, “Liver-Qi Stagnation” is a common visceral Qi Stagnation pattern. Systemic and visceral symptom manifestations show common overlaps (14).

Spleen-Qi Deficiency syndrome is characterized by features of are fatigue, asthenia, atrophied muscle, pale tongue with thin white coating and moderate, weak pulse in Spleen deficiency patients (15).

Qi Deficiency syndrome is mainly manifested by fatigue, shortness of breath or reluctance to speak, spontaneous sweating, a swollen tongue with tooth marks on the sides, and an insufficient, weak pulse; in women, menorrhagia is common in patients with Qi Deficiency (16).

Liver-Qi Stagnation syndrome patients mainly present with emotional problems, abdominal distension, hypochondriac pain, and a dark purple tongue (17).

Cold Coagulation syndrome is characterized by cold intolerance with a preference for warmth, and cold limbs (18).

Blood Stasis syndrome in gynecological diseases is mainly manifested by dysmenorrhea and menstrual clots (18).

Dampness is defined as feeling external dampness or abnormal water-liquid transportation in the body (19).

## 3 Results

### 3.1 Clinical characteristics of Chinese AM patients

The study included 1,816 AM patients, with 691 from low latitude region (LLR) and 1,125 from low latitude region (HLR). Among high-latitude patients, 652 were surveyed during the warm season and 473 during the cold season. The overall mean age was 39.56 ± 6.78 years, with 895 patients aged <40 years and 921 aged ≥ge years.

### 3.2 Symptom cluster characteristics by latitude

**Figure 1:**
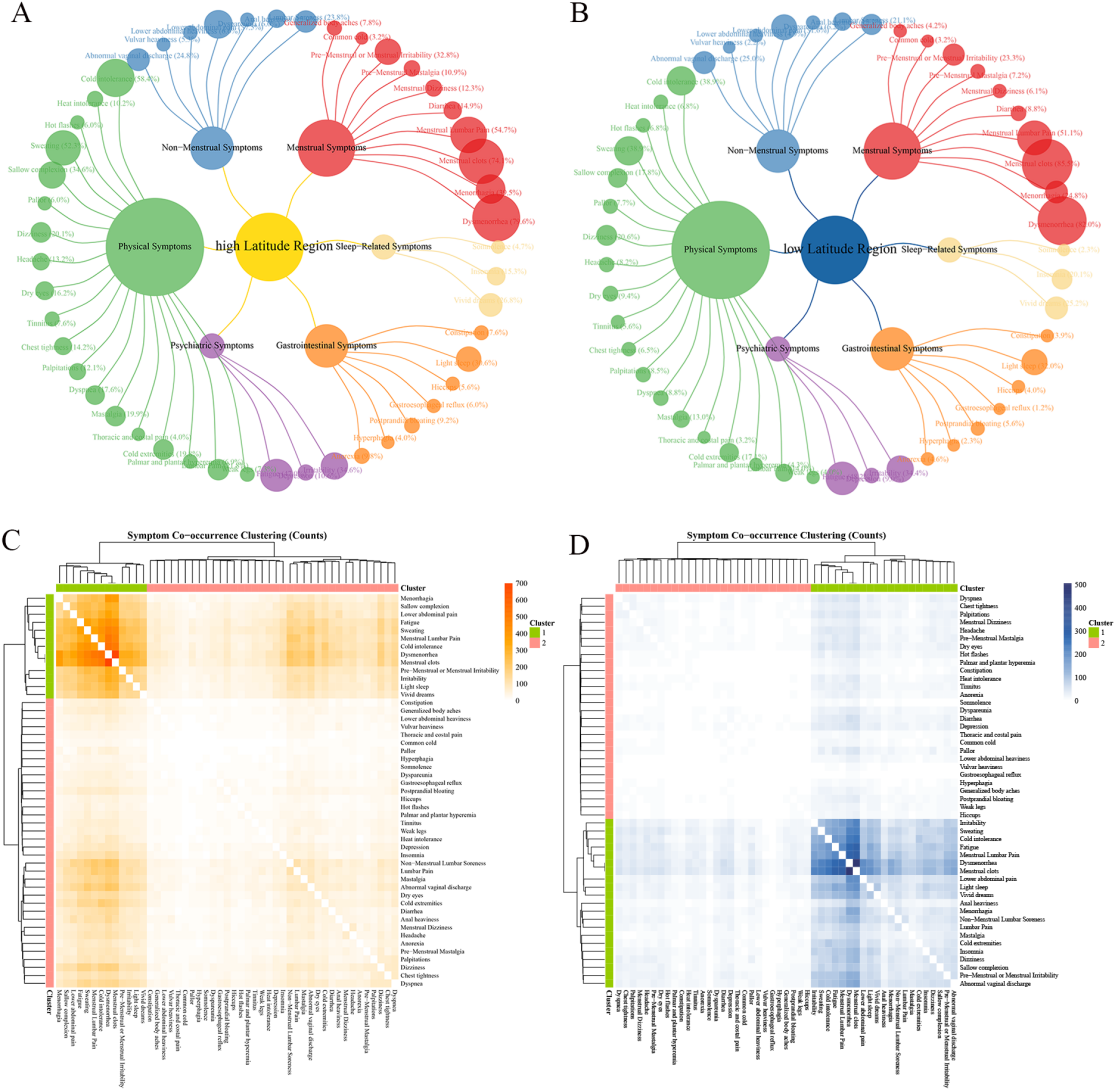
Characteristics of AM symptom groups at different latitudes in China. Ward hierarchical clustering was used in cluster analysis, and the number of clusters was 2. The color of heat map represents the co-occurrence frequency of symptoms, and the higher the co-occurrence frequency, the darker the color. **(A)** Frequency of six categories clinical symptoms in high latitude region; **(B)** Frequency of six categories clinical symptoms in low latitude region; **(C)** Culster1 was identified as the core symptom group in low latitudes region, and different orange shades indicated the frequency of simultaneous symptoms. **(D)** Culster1 was identified as the core symptom group in high latitudes region, and different blue shades indicated the frequency of simultaneous symptoms.

The mean age showed no significant difference between HLR (39.81 ± 6.79 years) and LLR (39.17 ± 6.77 years, *P*=0.052). Compared to low-latitude patients, high-latitude patients exhibited higher BMI (22.88 ± 3.11 vs. 22.05 ± 2.46 kg/m^2^, *P*<0.001), a greater proportion of postgraduate or above education (12% vs. 6.1%, P<0.001), and higher rates of monthly income >20,000 RMB (5.5% vs. 1.6%, P<0.001) (**Table 1**). A total of 49 clinical manifestations were identified across six symptom categories, with significant latitude-dependent variations (**Figures 1A, B**).

**Table 1:** Clinical characteristics of AM patients in different latitudes.

Variable	HLR (n=1125)	LLR (n=691)	P_value
Age (years)	39.81 ± 6.79	39.17 ± 6.77	0.052
Disease duration	32.22 ± 43.46	28.40 ± 38.71	0.051
Body mass index (BMI)	22.88 ± 3.11	22.05 ± 2.46	<0.001
Education level			<0.001
High school or below	321 (28.5%)	189 (27.4%)	
College	669 (59.5%)	460 (66.6%)	
Postgraduate or above	135 (12.0%)	42 (6.1%)	
Monthly income (RMB)			<0.001
<5,000	354 (31.5%)	276 (39.9%)	
5,000–20,000	709 (63.0%)	404 (58.5%)	
>20,000	62 (5.5%)	11 (1.6%)	
Occupational activity type			0.526
Heavy manual labor	21 (1.9%)	17 (2.5%)	
Moderate manual labor	163 (14.5%)	111 (16.1%)	
Light manual labor	440 (39.1%)	252 (36.5%)	
Sedentary work	501 (44.5%)	311 (45.0%)	
Obstetric history			0.624
No	372 (33.1%)	220 (31.8%)	
Yes	753 (66.9%)	471 (68.2%)	
Abortion history			0.077
No	513 (45.6%)	285 (41.2%)	
Yes	612 (54.4%)	406 (58.8%)	
Surgical history			0.566
No	898 (79.8%)	560 (81.0%)	
Yes	227 (20.2%)	131 (19.0%)	

**Figure 2:**
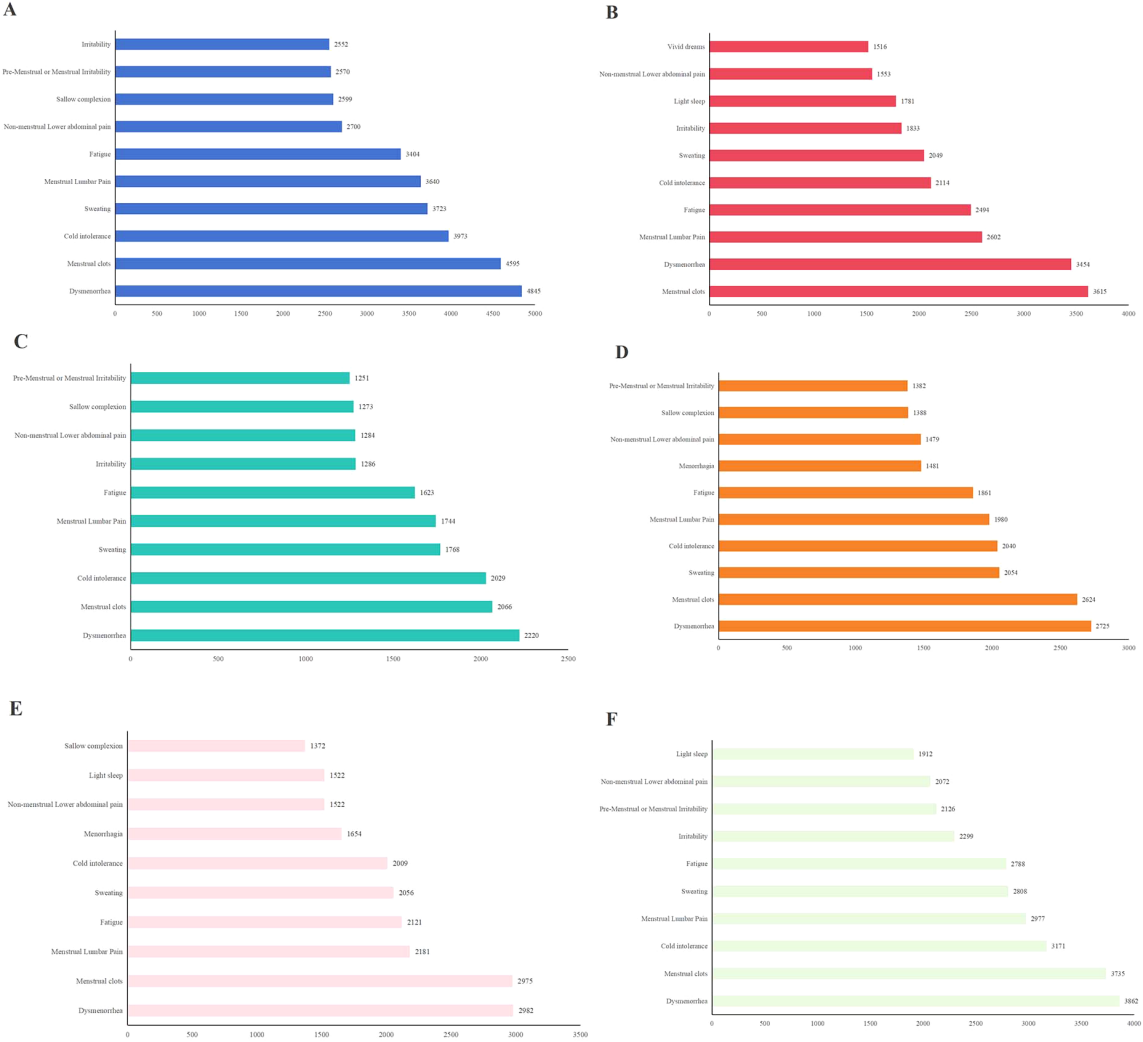
Co-occurrence times of top 10 core symptoms in different groups: **(A)** co-occurrence times of top 10 core symptoms in high latitudes region; **(B)** Co-occurrence times of top 10 core symptoms in low latitudes region; **(C)** Co-occurrence times of top 10 core symptoms in Cold season; **(D)** Co-occurrence times of top 10 core symptoms in Warm season; **(E)** Co-occurrence times of top 10 core symptoms in elder cases; **(F)** Co-occurrence times of top 10 core symptoms in young cases.

The results of the symptom co-occurrence matrix and hierarchical clustering analysis indicate that there were distinct high-frequency core symptom cluster in HLR (**Figure 1D**, **Figure 2A**) and LLR (**Figures 1C**, **2B**). Although most symptoms in the core symptom clusters of the two regions were similar, the associations between these core symptoms differed. In addition to the common menstrual symptoms that point to the “Blood Stasis syndrome”, the symptom of cold intolerance (HLR 58.4% vs. LLR 38.93%) has the highest number of connections with other symptoms in HLR, with a total of 3,973 occurrences. In LLR, the symptom of fatigue (HLR 47.02% vs. LLR 48.19%) has the highest number of associations with other symptoms, totaling 2,492 occurrences. Other symptoms include dysmenorrhea (HLR 79.56% vs. LLR 82.05%); menstrual clots (HLR 74.13% vs. LLR 85.53%); sweating (HLR 52.27% vs. LLR 38.93%); menstrual lumbar pain (HLR 54.67% vs. LLR 51.09%); non-menstrual lower abdominal pain (HLR 37.51% vs. LLR 31.55%); and irritability (HLR 34.58% vs. LLR 34.44%). Additionally, the core symptoms in high-latitude regions also include sallow complexion (HLR 34.58% vs. LLR 17.80%) and premenstrual or menstrual irritability (HLR 32.80% vs. LLR 23.30%), while low-latitude regions include light sleep (HLR 30.58% vs. LLR 31.98%) and vivid dreams (HLR 26.84% vs. LLR 25.18%).

Notably, high-latitude patients reported higher rates of cold-type abdominal pain during menstruation (HLR 25.60% vs. LLR 16.21%) and non-menstrual periods (HLR 7.29% vs. LLR 3.04%), consistent with TCM “Cold Congelation syndrome” pathophysiology.

The distribution difference of lesion types in different latitudes was analyzed (**Supplementary Table 1**). The results showed that there was no significant difference in the types of lesions in patients with different latitudes (P > 0.05).

### 3.3 Symptom cluster characteristics of AM patients in different seasons in high-latitude regions

**Figure 3:**
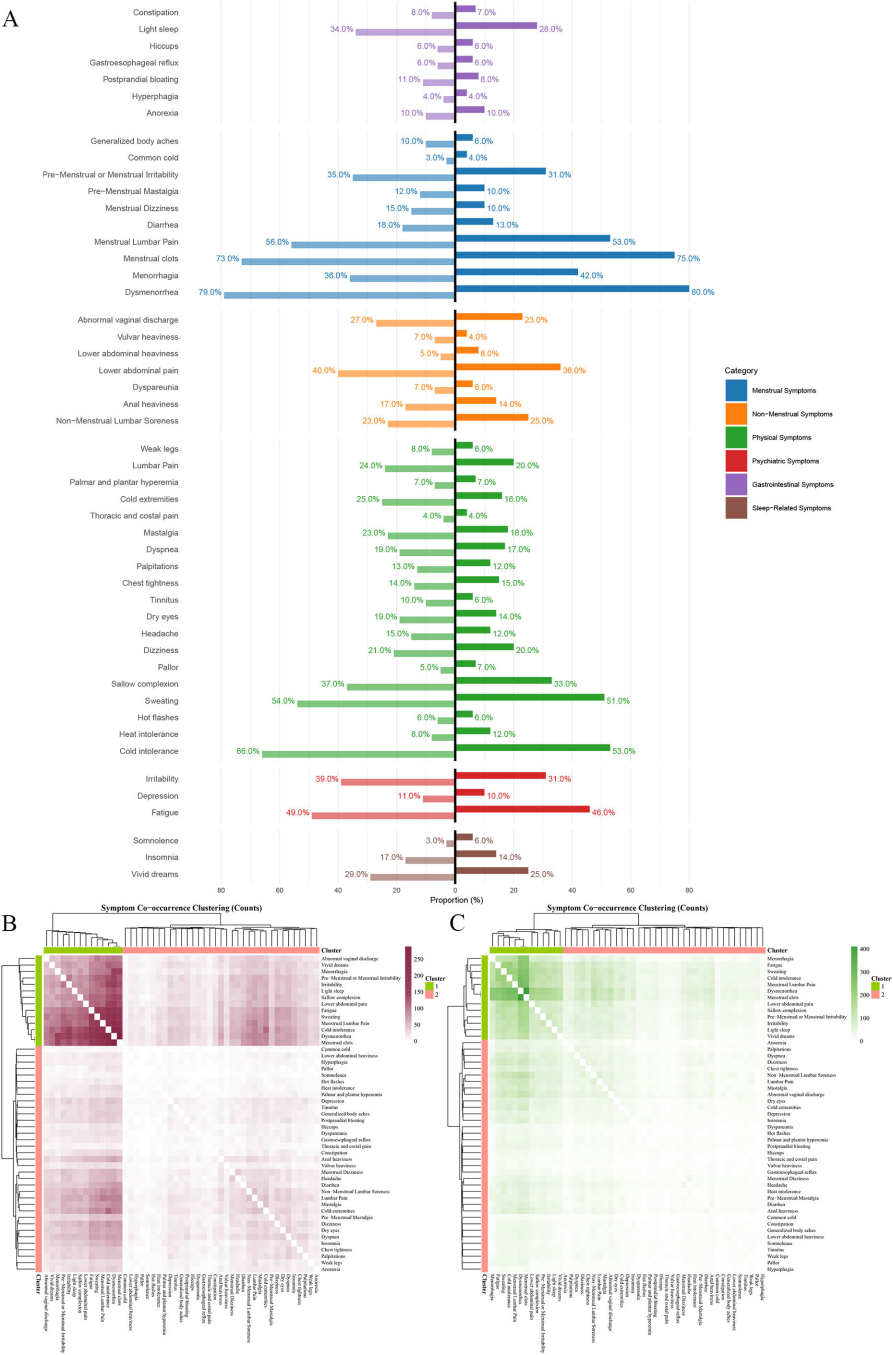
Characteristics of AM symptom groups at different seasons in China. Ward hierarchical clustering was used in cluster analysis, and the number of clusters was 2. The color of heat map represents the co-occurrence frequency of symptoms, and the higher the co-occurrence frequency, the darker the color. **(A)** Frequency of six categories clinical symptoms in cold season(left) and warm season(right); **(B)** Culster1 was identified as the core symptom group in cold season, and different purple shades indicated the frequency of simultaneous symptoms. **(C)** Culster1 was identified as the core symptom group in warm season, and different green shades indicated the frequency of simultaneous symptoms.

Among the patients included in HLR, the average age during the warm season was 40.15 ± 6.75 years, while the average age of patients during the cold season was 39.33 ± 6.81 years. There was a significant difference between the two groups (P = 0.044). Compared with the warm season, a higher proportion of patients during the cold season had a postgraduate or above degree or above (10.9% vs. 13.6%, P = 0.005), were engaged in sedentary labor (41.1% vs. 49.3%, P < 0.001), had a surgery history (17.0% vs. 24.5%, P = 0.002), and a smaller proportion of obstetric history (70.4% vs. 62.2%, P = 0.004). (**Table 2**) The influence of education level, type of work, surgical history, and childbirth history on core symptoms needs to be considered. The proportion of different symptoms in different seasons is shown in **Figure 3A**.

**Table 2:** Clinical characteristics of AM patients in different seasons in high latitudes.

Variable	Warm (n=652)	Cold (n=473)	P_value
Age (years)	40.15 ± 6.75	39.33 ± 6.81	0.044
Disease duration	31.36 ± 43.59	33.42 ± 43.29	0.431
Body mass index (BMI)	22.98 ± 3.03	22.73 ± 3.22	0.193
Education level			0.005
High school or below	210 (32.2%)	111 (23.5%)	
College	371 (56.9%)	298 (63.0%)	
Postgraduate or above	71 (10.9%)	64 (13.5%)	
Monthly income (RMB)			0.291
<5,000	207 (31.7%)	147 (31.1%)	
5,000–20,000	415 (63.7%)	294 (62.2%)	
20,000	30 (4.6%)	32 (6.8%)	
Occupational activity type			<0.001
Heavy manual labor	12 (1.8%)	9 (1.9%)	
Moderate manual labor	118 (18.1%)	45 (9.5%)	
Light manual labor	254 (39.0%)	186 (39.3%)	
Sedentary work	268 (41.1%)	233 (49.3%)	
Obstetric history			0.004
No	193 (29.6%)	179 (37.8%)	
Yes	459 (70.4%)	294 (62.2%)	
Abortion history			0.072
No	282 (43.3%)	231 (48.8%)	
Yes	370 (56.7%)	242 (51.2%)	
Surgical history			0.002
No	541 (83.0%)	357 (75.5%)	
Yes	111 (17.0%)	116 (24.5%)	

The results of the symptom co-occurrence matrix and hierarchical clustering analysis indicate that there were distinct high-frequency core symptom clusters in the cold season (**Figures 3B**, **2C**) and the warm season (**Figures 3C**, **2D**). Although most symptoms in the core symptom clusters of the two seasons were similar, the associations between these core symptoms differed. In addition to the common menstrual symptoms that point to the “Blood Stasis syndrome”, the symptom of cold intolerance (Cold 66.38% vs. Warm 52.61%) had the highest number of connections with other symptoms in the warm season, with a total of 2,029 occurrences. In the warm season, the symptom of sweating (Cold 54.33% vs. Warm 50.77%) had the highest number of associations with other symptoms, totaling 2,054 occurrences. Other symptoms included dysmenorrhea (Cold 79.49% vs. Warm 79.60%); menstrual clots (Cold 72.94% vs. Warm 75.00%); cold intolerance (Cold 66.38% vs. Warm 52.61%); sweating (Cold 54.33% vs. Warm 50.77%); menstrual lumbar pain (Cold 56.45% vs. Warm 53.37%); fatigue (Cold 48.63% vs. Warm 45.86%); irritability (Cold 38.90% vs. Warm 31.44%); non-menstrual lower abdominal pain (Cold 39.75% vs. Warm 35.89%); sallow complexion (Cold 37.42% vs. Warm 32.52%); and premenstrual or menstrual irritability (Cold 35.31% vs. Warm 30.98%). In addition, the core symptoms in the cold season also included irritability (Cold 38.90% vs. Warm 31.44%), while the warm season included menorrhagia (Cold 42.02% vs. Warm 35.94%).

The distribution difference of lesion types in seasons was analyzed (**Supplementary Table 2**). The mixed type of lesion type was more in warm season(P=0.008). In addition, we analyzed whether the focus type was related to menorrhagia, a special symptom in warm season. Taking menorrhagia as the dependent variable, season as the independent variable and lesion type as the covariate, we found that season itself had no significant effect on menorrhagia (OR: 0.796, 95%CI:0.622-1.02, P=0.07) (**Supplementary Table 3**).

### 3.4 Symptom cluster characteristics of AM patients in different age groups

**Figure 4:**
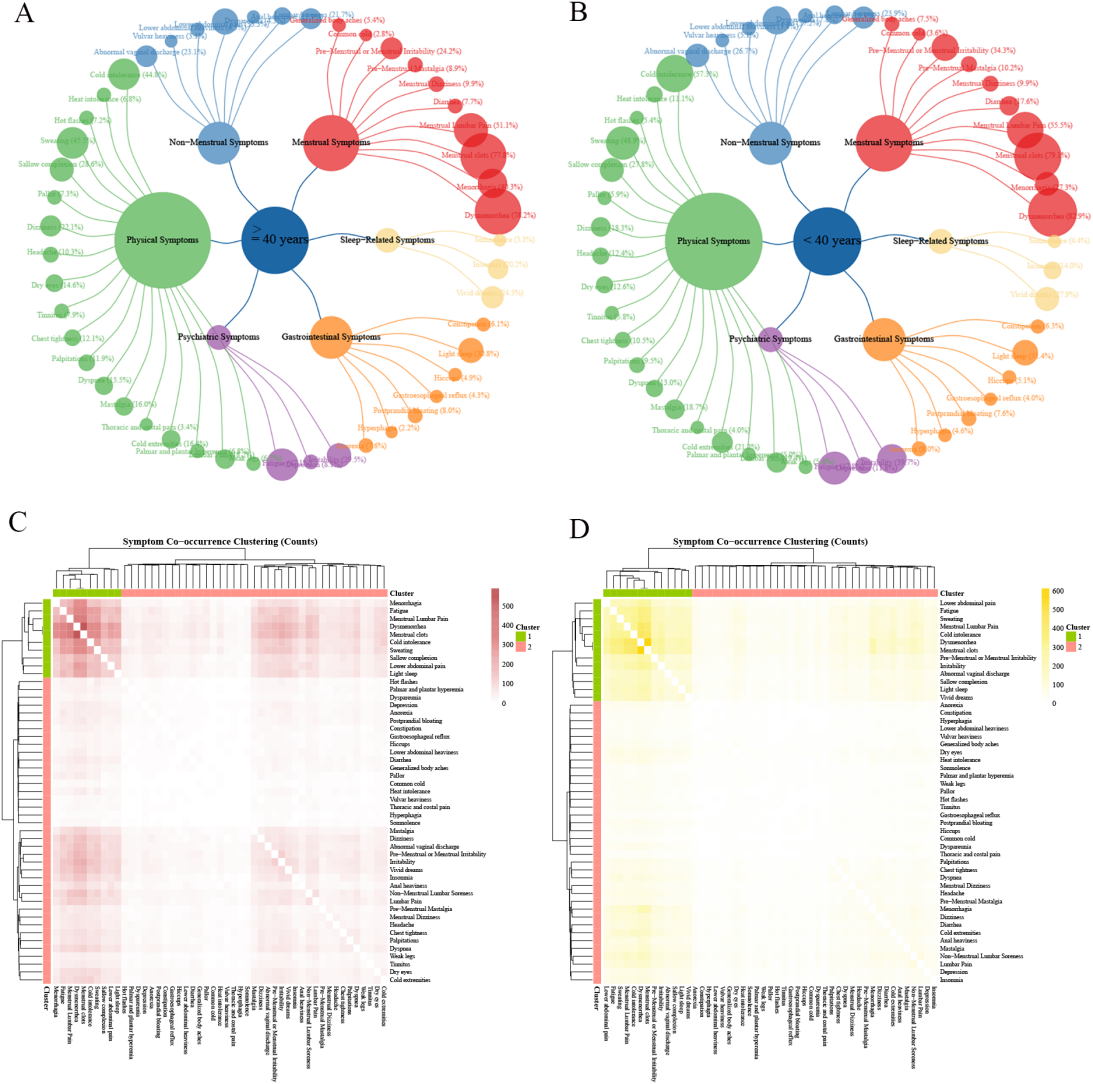
Characteristics of AM symptom groups at different age groups in China. Ward hierarchical clustering was used in cluster analysis, and the number of clusters was 2. The color of heat map represents the co-occurrence frequency of symptoms, and the higher the co-occurrence frequency, the darker the color. **(A)** Frequency of six categories clinical symptoms in elder cases; **(B)** Frequency of six categories clinical symptoms in young cases; **(C)** Culster1 was identified as the core symptom group in elder cases, and different red shades indicated the frequency of simultaneous symptoms. **(D)** Culster1 was identified as the core symptom group in young cases, and different yellow shades indicated the frequency of simultaneous symptoms.

The average age of patients under 40 years old included in the study was 33.95 ± 3.98 years, while the average age of patients aged 40 and above was 45.02 ± 3.86 years, with a significant difference between the two groups (P < 0.001). There were no significant differences in latitude (P = 0.904) and season (P = 0.058) among different age groups. Compared with AM patients aged 40 and above, those under 40 had a shorter disease duration, lower BMI, and a higher proportion of individuals with a postgraduate degree or above (15.4% vs. 4.2%, P < 0.001). A greater proportion of younger patients were engaged in sedentary labor (51.7% vs. 37.9%, P < 0.001), and a smaller proportion of obstetric history (40.8% vs. 86.2%, P < 0.001). (**Table 3**) The influence of education level, type of work, surgical history, and obstetric history on core symptoms needs to be considered. In terms of treatment goals, patients under 40 had a more pronounced demand for fertility, with a higher proportion aiming for “assisted pregnancy” (29.2% vs. 6.5%, P < 0.001), while patients aged 40 and above were more likely to aim for “reducing menstrual flow” (27.0% vs. 15.4%, P < 0.001) and “regulating menstrual cycle” (18.2% vs. 13.3%, P < 0.001). (**Table 4**) The proportion of different symptoms in different age groups is shown in **Figures 4A, B**.

**Table 3:** Clinical characteristics of AM patients in different age groups.

Variable	<40 years(n=895)	≥40 years(n=921)	P_value
Age (years)	33.95 ± 3.98	45.02 ± 3.86	<0.001
Disease duration	25.23 ± 34.61	36.15 ± 47.07	<0.001
Body mass index (BMI)	21.99 ± 2.92	23.12 ± 2.79	<0.001
Region			0.904
high latitudes	468 (52.3%)	478 (51.9%)	
low latitudes	427 (47.7%)	443 (48.1%)	
Season			0.558
Warm	334 (37.3%)	357 (38.8%)	
Cold	561 (62.7%)	564 (61.2%)	
Education level			<0.001
High school or below	127 (14.2%)	383 (41.6%)	
College	630 (70.4%)	499 (54.2%)	
Postgraduate or above	138 (15.4%)	39 (4.2%)	
Monthly income (RMB)			<0.001
<5,000	148(38.44%)	376 (40.8%)	
5,000–20,000	224(58.18%)	512 (55.6%)	
20,000	13(3.38%)	33 (3.6%)	
Occupational activity type			<0.001
Heavy manual labor	7 (0.8%)	31 (3.4%)	
Moderate manual labor	80 (8.9%)	194 (21.1%)	
Light manual labor	345 (38.5%)	347 (37.7%)	
Sedentary work	463 (51.7%)	349 (37.9%)	
Obstetric history			<0.001
No	465 (52.0%)	127 (13.8%)	
Yes	430 (48.0%)	794 (86.2%)	
Abortion history			<0.001
No	497 (55.5%)	301 (32.7%)	
Yes	398 (44.5%)	620 (67.3%)	
Surgical history			0.097
No	704 (78.7%)	754 (81.9%)	
Yes	191 (21.3%)	167 (18.1%)	

**Table 4:** Treatment needs of different age groups.

Treatment needs	<40 years(n=895)	≥40 years(n=921)	P_value
Relieve pain			0.09
No	333 (37.2%)	379 (41.2%)	
Yes	562 (62.8%)	542 (58.8%)	
Reduce menstrual flow			<0.001
No	757 (84.6%)	672 (73.0%)	
Yes	138 (15.4%)	249 (27.0%)	
Adjust menstrual cycle			<0.001
No	776 (86.7%)	753 (81.8%)	
Yes	119 (13.3%)	168 (18.2%)	
Assisted pregnancy			<0.001
No	634 (70.8%)	861 (93.5%)	
Yes	261 (29.2%)	60 (6.5%)	
Control focus			0.370
No	504 (56.3%)	539 (58.5%)	
Yes	391 (43.7%)	382 (41.5%)	
Prevent recurrence			0.300
No	788 (88.0%)	795 (86.3%)	
Yes	107 (12.0%)	126 (13.7%)	

The results of the symptom co-occurrence matrix and hierarchical clustering analysis indicated that there were distinct high-frequency core symptom clusters in elder patients (**Figures 4C**, **2E**) and younger patients (**Figures 4D**, **2F**). Although most symptoms in the core symptom clusters of the two age groups were similar, the associations between these core symptoms differed. In addition to the common menstrual symptoms that point to the “Blood Stasis syndrome”, the symptom of cold intolerance (Young 66.38% vs. Elder 52.61%) had the highest number of connections with other symptoms in younger patients, with a total of 3,171 occurrences. In elder patients, the symptom of fatigue (Young 47.12% vs. Elder 47.82%) had the highest number of associations with other symptoms, totaling 2,121 occurrences. Other symptoms included dysmenorrhea (Young 82.91% vs. Elder 78.18%); menstrual clots (Young 79.11% vs. Elder 77.85%); menstrual lumbar pain (Young 55.53% vs. Elder 51.14%); fatigue (Young 47.82% vs. Elder 47.12%); sweating (Young 48.94% vs. Elder 45.49%); cold intolerance (Young 57.32% vs. Elder 44.84%); non-menstrual lower abdominal pain (Young 37.21% vs. Elder 33.33%); light sleep (Young 31.40% vs. Elder 30.84%). In addition, core symptoms in younger patients also included irritability (Elder 29.53% vs. Young 39.66%) and premenstrual or menstrual irritability (Elder 24.21% vs. Young 34.30%), while older patients included menorrhagia (Young 27.26% vs. Elder 40.28%) and sallow complexion (Young 27.82% vs. Elder 28.56%).

The distribution difference of lesion types in age groups was analyzed (**Supplementary Table 4**). There were significant differences in the types of lesions in patients of different ages, and the proportion of fixed lesion type is higher in elderly patients(P=0.002). We also analyzed the influence of lesion types on the symptoms of AM patients at different age groups (dysmenorrhea, menorrhagia and non-menstrual minor abdominal pain as dependent variables, age as independent variable and lesion types as covariate). The results show that being over 40 years old was still a risk factor for menorrhagia(OR:1.75, 95CI%:1.43-2.14, P<0.001) and a protective factor for dysmenorrhea(OR: 0.688, 95CI%:0.541-0.872, P<0.002) (**Supplementary Figure 1**), which was consistent with the previous results. More importantly, compared with diffuse type, mixed type is an independent risk factor for dysmenorrhea, menorrhagia and non-menstrual minor abdominal pain(OR>1, P<0.05). The overall sample analysis showed that the clinical symptoms of mixed type of AM were more significant (**Supplementary Figure 2**).

### 3.5 Treatment of different dimensions

We analyzed the characteristics of treatment history across different dimensions and their impact on symptoms. The results showed that there were no significant differences in the history of Western medicine treatment across different latitudes, as well as in the overall treatment history across different seasons and age groups (P > 0.05) (**Supplementary Tables 5**, **7**, **8**). However, there are significant differences in the treatment history of traditional Chinese medicine at different latitudes (**Supplementary Table 5**). Therefore, we further included treatment history as a covariate in the equation analyzing the impact of latitude on cold intolerance. (**Supplementary Table 6**) The results found that high latitude remains a risk factor for cold intolerance (OR:2.29, 95%CI:1.88-2.79, P<0.001); meanwhile, having a history of TCM treatment is also a risk factor for cold intolerance (OR:1.26, 95%CI:1.04-1.54, P=0.021). In addition, when analyzing the correlation between treatment history and AM-specific symptoms in the overall sample, we found that patients who had sought drug treatment before and revisited the outpatient clinic had a higher prevalence of dysmenorrhea (**Supplementary Figure 2**).

We analyzed the selection of treatment methods across groups after the questionnaire survey. Compared with the HLR, a significantly higher proportion of patients in the LLR opted for TCM decoction treatment (87.3% vs. 67.3%, P < 0.001). Younger patients showed a higher rate of choosing TCM decoction treatment than elder patients (81.7% vs. 68.3%, P < 0.001). No statistical differences were observed in other inter-group comparisons (P > 0.05) (**Table 5**).

**Table 5:** Selection of treatment methods.

Group	Western medicine			Traditional Chinese medicine decoction		
	No	Yes	P	No	Yes	P
LLR	629 (91.0%)	62 (9.0%)	0.322	88 (12.74%)	603 (87.3%)	<0.001
HLR	1008 (89.6%)	117 (10.4%)		368 (32.71%)	757 (67.3%)	
Warm	582 (89.3%)	70 (10.7%)	0.665	216 (33.1%)	436 (66.9%)	0.726
Cold	426 (90.1%)	47 (9.9%)		152 (32.1%)	321 (67.9%)	
<40 years	817 (91.3%)	78 (8.7%)	0.108	164 (18.3%)	731 (81.7%)	<0.001
≥40 years	820 (89.0%)	101 (11.0%)		292 (31.7%)	629 (68.3%)	

To validate whether the syndrome differentiation model aligns with clinical medication practices, according to a small number of patients who have information about the choice of Chinese medicines, we first determined four core Chinese herbs pairs: Warm Yang and Disperse Cold Pair: Cinnamomum cassia Twig - Carbonized Ginger (Guizhi - Paojiang); Invigorate Spleen and Supplement Qi Pair: Astragalus Root - Codonopsis Root (Huangqi - Dangshen);Soothe Liver and Regulate Qi Pair: Bupleurum Root - Cyperus Rhizome (Chaihu - Xiangfu);Supplement Qi and Nourish Blood Pair: Astragalus Root - Angelica Sinensis Root (Huangqi - Danggui), and counted their applications. As shown in **Table 6**, the application proportion of the Warm Yang and Disperse Cold Pair is higher in HLR (94.5% vs. 5.50%) and cold seasons (60.19% vs. 39.81%), while the application proportion of the Invigorate Spleen and Supplement Qi Pair is higher in LLR (67.27% vs. 32.73%). The Warm Yang and Disperse Cold Pair and Soothe Liver and Regulate Qi Pair were more frequently used in younger patients (57.80% vs. 42.20%, 68.89% vs. 31.11%), whereas the Supplement Qi and Nourish Blood Pair was more commonly applied in elder patients (71.43% vs. 44.44%).

**Table 6:** Application of core Chinese medicine pairs.

Group	Cinnamomum cassia Twig - Carbonized Ginger		Astragalus Root - Codonopsis Root		Bupleurum Root - Cyperus Rhizome		Astragalus Root - Angelica Sinensis Root	
	n	%	n	%	n	%	n	%
LLR	6	5.50%	74	67.27%	3	6.67%	41	65.08%
HLR	103	94.50%	36	32.73%	42	93.33%	32	50.79%
Warm	41	39.81%	17	47.22%	21	50.00%	20	31.75%
Cold	62	60.19%	19	52.78%	21	50.00%	12	19.05%
<40 years	63	57.80%	64	41.82%	31	68.89%	45	44.44%
≥40 years	46	42.20%	46	58.18%	14	31.11%	28	71.43%

## 4 Discussion

This cross-sectional study investigated the current status of AM diagnosis and treatment in China from 2020 to 2022. We extracted baseline characteristics and clinical symptom data from questionnaires and performed stratified analyses based on the TCM theory of “Treatment in Accordance with Three Categories of Etiologic Factors”, examining spatial (LLR vs HLR), temporal (warm vs cold season), and age (<40 vs ≥s4 years) distributions of AM patients’ clinical characteristics and symptom clusters. This research aims to establish data-driven support for TCM syndrome differentiation in AM, transforming previously vague and subjective diagnostic approaches into more objective and precise methods, thereby facilitating accurate treatment and medication decisions. Our results demonstrate variations in clinical symptom prevalence, symptom cluster features, and core symptoms across different strata, while consistently highlighting “Blood Stasis syndrome” as the fundamental pathological mechanism. Meanwhile, in the spatial dimension, the syndrome of Cold Coagulation and Blood Stasis is the main syndrome in HLR. In LLR, the syndrome of Spleen-Qi Deficiency and Blood Stasis is the main syndrome. In the temporal dimension, the “heen Blood Stasis with syndromen is prominent in the cold season,; In warm season, the syndrome of dampness is more common. In the age dimension, young patients are mostly characterized by “cold and blood stasis combined with liver depression”. The elderly patients mainly suffer from “Qi deficiency and blood stasis syndrome”. These findings provide scientific evidence supporting the diagnostic application of the TCM theory of “Treatment in Accordance with Three Categories of Etiologic Factors” in AM. Besides, the clinical value of the three-dimensional syndrome differentiation model established in this study mainly lies in: providing key directions for diagnosis and treatment in different regions (such as giving priority to warming Yang and dispelling cold in HLR); Guide the adjustment of seasonal treatment strategy (strengthening warming Yang in cold season); Achieve accurate medication by age (young patients cooperate with soothing the liver and regulating Qi, and elderly patients focus on supplementing Qi). These findings promote the traditional syndrome differentiation from static classification to dynamic individualized treatment plan generation system.

When examining spatial factors, no significant difference in age distribution was observed between HLR and LLR (P=0.052), indicating comparable population characteristics. From the perspective of TCM syndrome, the HLR exhibited a symptom network characterized by cold intolerance (association frequency: 3973) as the core manifestation. Given that HLR experience lower temperatures than LLR, our findings align with the TCM pathogenesis theory that cold regions are more susceptible to invasion by Cold Pathogens (20). Previous syndrome surveys demonstrated that a prevalence of “Cold Coagulation and Blood Stasis syndrome” was higher in northern regions than other regions (21). Concurrently, the HLR showed a doubled incidence of sallow complexion (34.58% vs 17.80%), which may be associated with the higher prevalence of menorrhagia in these patients (39.47% vs 24.75%). In contrast, the low-latitude group’s symptom cluster structure, centered on “fatigue” (association frequency: 2492), closely corresponds to the syndrome pattern of “Spleen-Qi Deficiency and Blood Stasis” The prominent manifestation of mental symptoms such as light sleep (31.98% vs 30.58%) and vivid dreaming (25.18% vs 26.84%) in this group may relate to the disturbance of mental activity caused by dampness accumulation. Although both regions shared irritability as a core symptom (38.90% vs 31.44%), the HLR additionally exhibited premenstrual or menstrual irritability at a significantly higher rate (32.80% vs 23.30%). Notably, the HLR demonstrated significantly higher proportions of both higher education level (12% vs 6.1%) and income level (5.5% vs 1.6%). This demographic pattern reflects the economic disparities documented in the “Notice of the General Office of the State Council on Printing and Distributing the Reform Plan for the Division of Central and Local Financial Powers and Expenditure Responsibilities in the Healthcare Sector,” which indicates that the average economic tier of the primary provinces and cities in our high-latitude study areas exceeds that of low-latitude regions.

These findings suggest that symptom reporting was confounded by regional economic factors. To verify this theory, we took irritability and premenstrual or menstrual irritability as outcome indicators, and included these factors as covariates in the latitude, season and population models respectively, confirming that high education, high income and sedentary work are closely related to emotional symptoms in AM patients. (**Supplementary Figure 3**) From a sociocultural perspective, research established positive correlations between higher education levels and increased risks of obsessive-compulsive disorder, bipolar disorder, and anxiety disorders (22). Higher-income populations typically experience greater occupational stress, and the productivity losses associated with AM may exacerbate psychological stress responses. Study demonstrated associations between chronic stress and conditions including chronic pelvic pain, anxiety, and depression (23, 24), which explained the co-occurrence of higher incidence of non-menstrual lower abdominal pain (37.51% vs 31.55%) and emotional symptoms in the high-latitude group.

Patients with AM in HLR exhibit the syndrome characteristics of “Cold Congelation and Blood Stasis”. To further clarify whether there are significant seasonal dynamic changes, we shifted the focus to temporal factors. The results showed that patients in the cold season exhibited more pronounced syndrome characteristics of “Cold Congelation and Blood Stasis”. The incidence of cold intolerance was higher in the cold season than in the warm season (66.38% vs. 52.61%), and it had the strongest association with the symptom network (2,029 times), which is in line with the TCM pathogenesis theory. In contrast, patients in the warm season had the highest association with sweating (2,054 times), which may reflect the pathogenesis of “Dampness”. Even in the warm season, HLR still retained a certain “Cold Coagulation” than LLR (52.61% vs. 38.93%). The study proposed that in winter, the short daylight hours and low average temperatures make women more prone to qi stagnation syndrome (25), which is consistent with our finding that irritability is one of the core symptoms in the cold season. It should be noted that patients in the cold season were younger (39.33 ± 6.81 vs. 40.15 ± 6.75 years, P = 0.044), and the proportion of surgical history and nulliparity was higher (24.5% vs. 17.0% and 62.2% vs. 70.4%, respectively), which may influence the interpretation of the syndrome. After correcting for confounding factors such as surgical history and parity (**Supplementary 1**), we found that childbirth is a protective factor for AM pain but a risk factor for menorrhagia, which again explains why the proportion of menorrhagia in older patients is higher than that in younger patients, while the proportion of dysmenorrhea and non-menstrual abdominal pain is lower than that in younger patients. Disease duration is a risk factor for dysmenorrhea and non-menstrual abdominal pain in adenomyosis. Surgical history was not significantly associated with symptoms of adenomyosis. For example, younger age and patient who never had a child lead to non-menstrual pelvic pain symptoms (26) (cold 39.75% vs. warm 35.89%).

This study also revealed the dynamic evolution of the symptom spectrum of AM patients with age. Younger patients (<40 years) exhibited a significant “Cold Congelation and Blood Stasis with Liver Qi Stagnation” composite syndrome: the incidence of cold intolerance was significantly higher (57.32% vs. 44.84%), and cold intolerance had the highest association with the symptom network (3,171 times). Emotional symptoms were prominent, with irritability (39.66% vs. 29.53%) and premenstrual or menstrual irritability (34.30% vs. 24.21%) about 10% higher than in the elder group. The core symptoms in the elder group did not include irritability or premenstrual or menstrual irritability. Older patients (ati years) exhibited the syndrome characteristics of “Qi Deficiency and Blood Stasis”: the incidence of menorrhagia increased (40.28% vs. 27.26%); fatigue became the core hub symptom (2,121 times), which is consistent with the TCM view that during the perimenopausal period, the body’s qi and blood gradually decline. The higher proportion of nulliparity in the younger group (62.2% vs. 70.4%, P = 0.004) and more surgical history in the older group (24.5% vs. 17.0%, P = 0.002) are also consistent with the basic characteristics of AM women in different age groups. Education level, monthly income, and occupational form also increased the risk of Liver Qi Stagnation in younger patients. The study found that patients with Liver Qi Stagnation syndrome who had higher education levels and a monthly income of more than 5,000 yuan accounted for a higher proportion (27). These individuals mostly engaged in mental work with relatively less physical activity, making them more prone to liver qi stagnation syndrome (27). In addition, younger patients are mostly in their childbearing years with significant fertility demands. Infertility itself and uncertainty about treatment methods can cause significant mental stress (28). The study found that infertility related to endometriosis is a powerful trigger for depression in women (29). Since younger age and surgical history are risk factors for AM pain symptoms, this explains why younger patients in this study mostly sought medical treatment to relieve pain. The study reported that approximately 90% of women experience menstrual disorders during the perimenopausal period (30), which is consistent with our finding that older patients mostly aimed to regulate their menstrual cycles as a treatment goal. Menorrhagia is also one of the common symptoms during the perimenopausal period (31). From the perspective of TCM theory, “treatment of the elderly focuses on the spleen”, older patients have Qi deficiency, and the Qi fails to control blood, hence the occurrence of menstrual disorders or menorrhagia.

This study systematically revealed the distribution patterns and influencing factors of TCM syndromes in AM through multidimensional symptom cluster analysis, and obtained the following important findings: (1) In terms of theoretical validation: it confirmed the scientific value of the TCM theory of “Treatment in Accordance with Three Categories of Etiologic Factors” For the first time, it proved through a large sample of data that AM has significant geographical latitude gradient differences (Syndrome of Cold Congelation vs. Spleen Deficiency, seasonal dynamic changes (more severe cold symptoms in winter and dampness in summer), and age evolution patterns (Liver Qi Stagnation in youth and Qi Deficiency in middle age). All stratifications maintained “Blood Stasis” as the core pathogenesis, providing an evidence-based basis for the basic treatment principle of activating blood circulation and removing blood stasis in AM. (2) The clinical value of the three-dimensional syndrome differentiation model established in this study mainly lies in providing key guidance for diagnosis and treatment at different levels. These findings have driven the transformation of traditional syndrome differentiation from static classification to dynamic personalized treatment plan generation systems. This study also has the following limitations: (1) Confounding factors were not controlled: the influence of regional socioeconomic levels and patients’ medical history and treatment history on symptoms and syndromes still need further research; (2) The cross-sectional design limits causal inference; (3) The classification of seasons as warm is a simple setting based on the geographical environment in China, lacking objective records of environmental parameters (such as specific temperature and humidity data).

The innovative value of this study lies in combining traditional TCM theory with modern data mining technology to establish a “space-time-life” three-dimensional syndrome differentiation model for AM symptoms, providing an important paradigm for promoting precise diagnosis and treatment in TCM gynecology. Future studies should conduct multicenter prospective cohort validation and explore the association mechanisms between symptom clusters, biological markers, and genetic polymorphisms.

## Data Availability

The raw data supporting the conclusions of this article will be made available by the authors, without undue reservation.
