# Supplementary material for: Spatial, temporal and demographic distribution characteristics of adenomyosis symptom clusters from the perspective of traditional Chinese medicine: a multicenter cross-sectional study in China from 2020 to 2022
Source: Front Endocrinol (Lausanne). 2025 Aug 7;16:1605310. doi: 10.3389/fendo.2025.1605310 (PMC12367498; doi:10.3389/fendo.2025.1605310)
Supplement: Supplementary file 1 [file DataSheet1.zip › Figure legends.docx]

**Supplement**

**Supplement 1:** Influence of past history and disease duration on the symptoms of AM patients.

**Supplement 2:** The difference of lesion type in different groups of AM patients and its influence on symptoms

**Supplement Figure**:

**Figure legends:**

**Supplement Figure 1** Effect of lesion types on AM symptoms in different age groups

**Supplement Figure 2** Effect of lesion types and medication history on the specific symptoms of AM patients.

**Supplement Figure 3** Influence of social factors on emotional symptoms

A: Influence of social factors at different latitudes on irritability symptom

B: Influence of social factors at different latitudes on premenstrual or menstrual irritability symptom

C: Influence of social factors at different seasons on irritability symptom

D: Influence of social factors at different season on premenstrual or menstrual irritability symptom

E: Influence of social factors at different age group on irritability symptom

F: Influence of social factors at different age group on premenstrual or menstrual irritability symptom
