# Supplementary figures and images for: Spatial, temporal and demographic distribution characteristics of adenomyosis symptom clusters from the perspective of traditional Chinese medicine: a multicenter cross-sectional study in China from 2020 to 2022
Source: Front Endocrinol (Lausanne). 2025 Aug 7;16:1605310. doi: 10.3389/fendo.2025.1605310 (PMC12367498; doi:10.3389/fendo.2025.1605310)

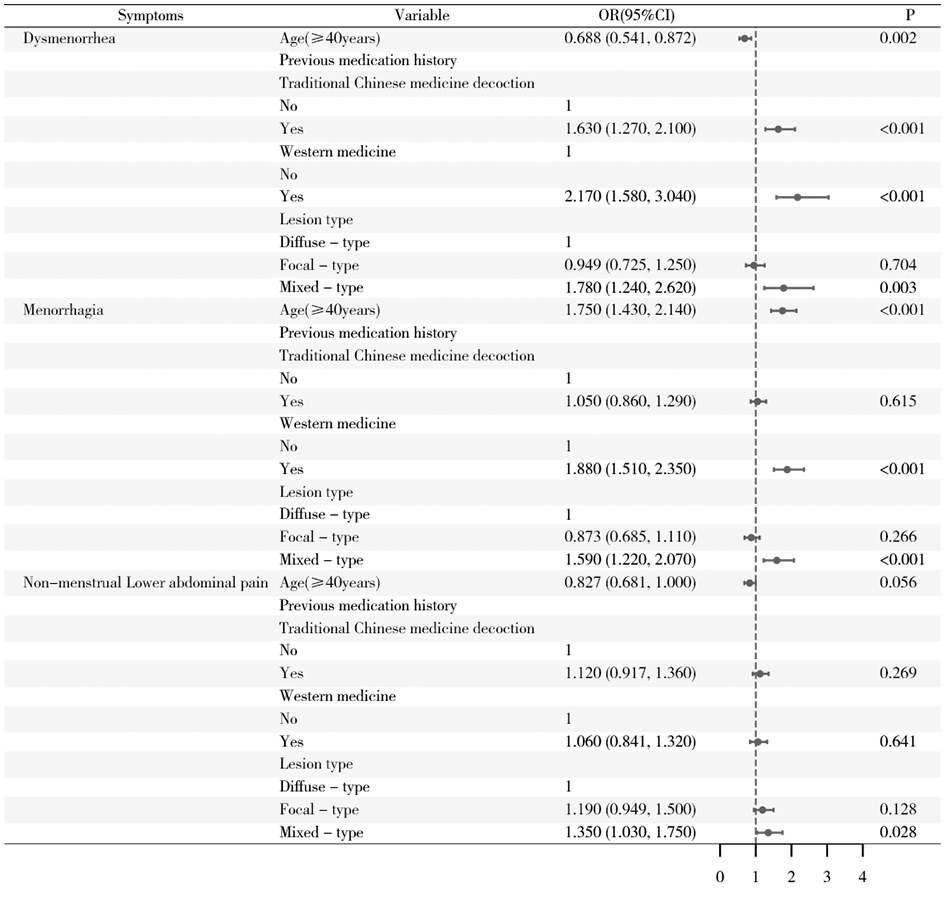

Supplement: Supplementary file 1 [file DataSheet1.zip › Supplement Figure1.tif]

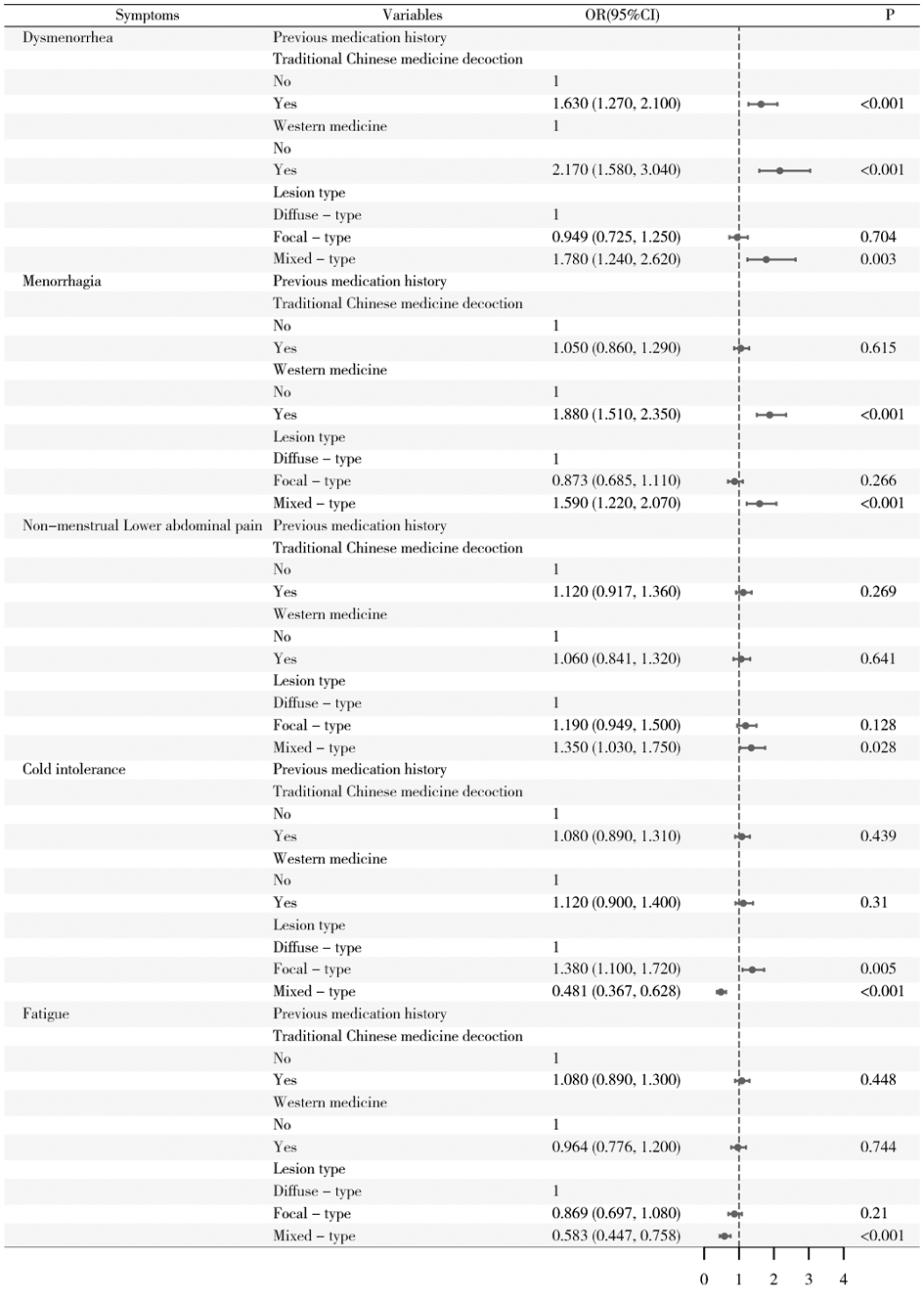

Supplement: Supplementary file 1 [file DataSheet1.zip › Supplement Figure2.tif]

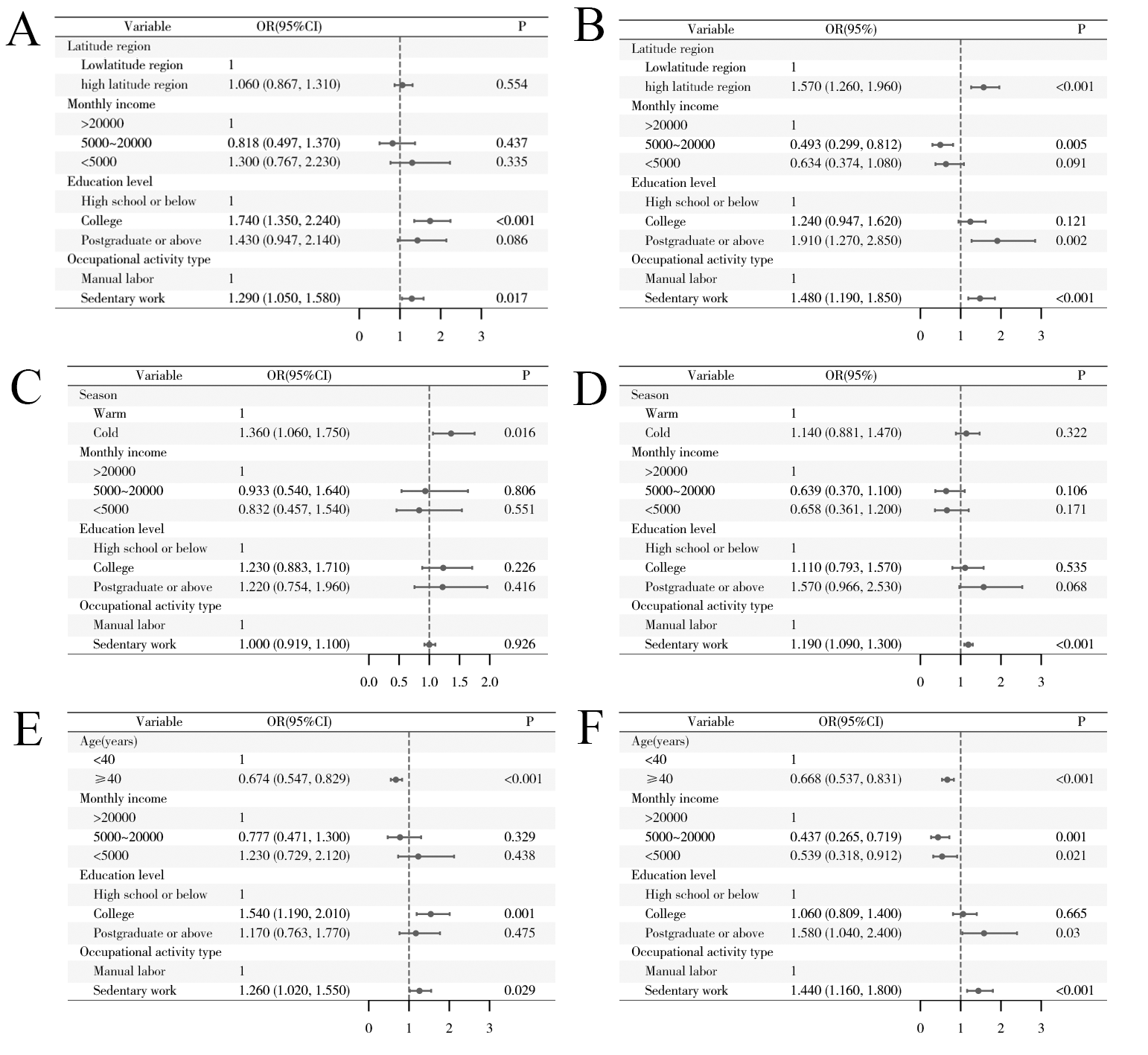

Supplement: Supplementary file 1 [file DataSheet1.zip › Supplement Figure3.tif]
